# Supplementary material for: Saccharomyces boulardii (CNCM I-745) improves intestinal damage in sepsis by remodeling intestinal flora structure
Source: Front Cell Infect Microbiol. 2025 Jul 31;15:1602792. doi: 10.3389/fcimb.2025.1602792 (PMC12350329; doi:10.3389/fcimb.2025.1602792)
Supplement: Supplementary file 1 [file DataSheet1.zip › summary/summary/7_Differential_analysis/3_Indicator_result/5_Family/Family_indicator_Group.pdf]

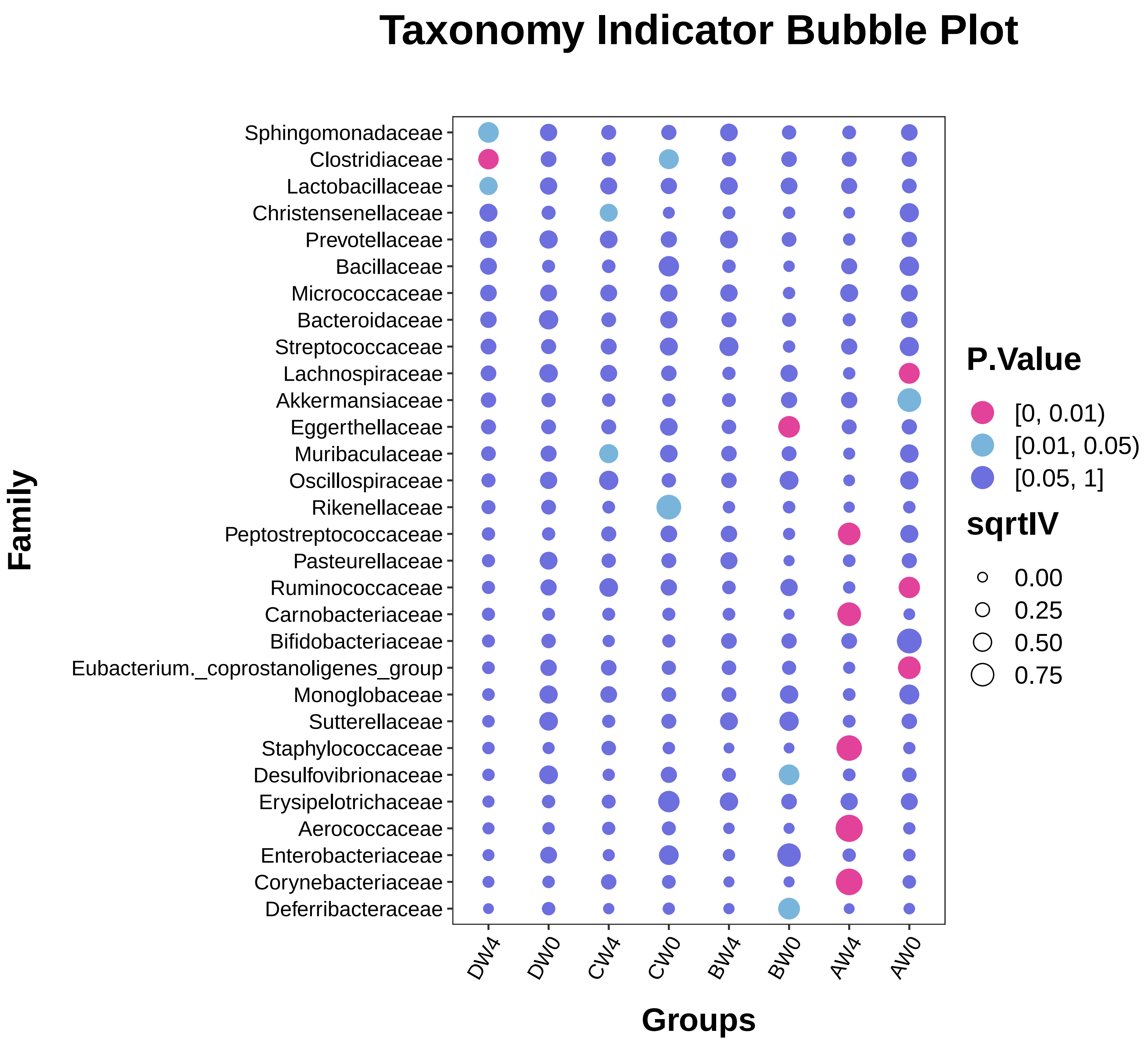
Taxonomy Indicator Bubble Plot
Family
Sphingomonadaceae
Clostridiaceae
Lactobacillaceae
Christensenellaceae
Prevotellaceae
Bacillaceae
Micrococcaceae
Bacteroidaceae
Streptococcaceae
Lachnospiraceae
Akkermansiaceae
Eggerthellaceae
Muribaculaceae
Oscillospiraceae
Rikenellaceae
Peptostreptococcaceae
Pasteurellaceae
Ruminococcaceae
Carnobacteriaceae
Bifidobacteriaceae
Eubacterium._coprostanoligenes_group
Monoglobaceae
Sutterellaceae
Staphylococcaceae
Desulfovibrionaceae
Erysipelotrichaceae
Aerococcaceae
Enterobacteriaceae
Corynebacteriaceae
Deferribacteraceae
DW4
DW0
CW4
CW0
BW4
BW0
AW4
AW0
Groups
P.Value
[0, 0.01)
[0.01, 0.05)
[0.05, 1]
sqrtIV
0.00
0.25
0.50
0.75
